# Precision medicine: preliminary results from the Initiative for Molecular Profiling and Advanced Cancer Therapy 2 (IMPACT2) study

**DOI:** 10.1038/s41698-021-00159-2

**Published:** 2021-03-19

**Authors:** Apostolia Maria Tsimberidou, David S. Hong, Siqing Fu, Daniel D. Karp, Sarina Piha-Paul, Merrill S. Kies, Vinod Ravi, Vivek Subbiah, Sunil M. Patel, Shi-Ming Tu, Filip Janku, John Heymach, Amber Johnson, Carrie Cartwright, Li Zhao, Jianhua Zhang, Donald A. Berry, David J. Vining, Andrew Futreal, Vincent A. Miller, Funda Meric-Bernstam

**Affiliations:** 1 The University of Texas MD Anderson Cancer Center, Department of Investigational Cancer Therapeutics, Houston, TX USA; 2 The University of Texas MD Anderson Cancer Center, Department of Thoracic, Head and Neck Medical Oncology, Houston, TX USA; 3 The University of Texas MD Anderson Cancer Center, Department of Sarcoma Medical Oncology, Houston, TX USA; 4 The University of Texas MD Anderson Cancer Center, Regional Cancer Center in Katy, TX; current affiliation: Kelsey Seybold Cancer Center, Houston, TX USA; 5 The University of Texas MD Anderson Cancer Center, Department of Genitourinary Medical Oncology, Houston, TX USA; 6 The University of Texas MD Anderson Cancer Center, Sheikh Khalifa Bin Zayed Al Nahyan Institute for Personalized Cancer Therapy, Houston, TX USA; 7 The University of Texas MD Anderson Cancer Center, Department of Genomic Medicine, Houston, TX USA; 8 The University of Texas MD Anderson Cancer Center, Department of Biostatistics, Houston, TX USA; 9 The University of Texas MD Anderson Cancer Center, Department of Abdominal Imaging, Houston, TX USA; 10 Foundation Medicine, Inc., Cambridge, MA USA

**Keywords:** Targeted therapies, Cancer genomics, Molecular medicine

## Abstract

Precision medicine is associated with favorable outcomes in selected patients with cancer. Herein, we report an interim analysis of IMPACT2, an ongoing randomized study evaluating genomic profiling and targeted agents in metastatic cancer. Patients with metastatic cancer underwent tumor genomic profiling (ClinialTrials.gov: NCT02152254), and 69 patients met the criteria for randomization. Tumor board and multidisciplinary review of molecular alterations optimized treatment selection. From 5/2014 to 4/2017, 320 patients (median age, 63 years; men, 47%) had tumor molecular aberrations, and 213 (66.56%) received anticancer therapy. The most frequently mutated genes were *TP53* (42%), *KRAS* (16%), *PIK3CA* (12%), and *CDKN2A* (11%). The median OS was 10.9 months (95% CI, 8.8–12.9). OS was shorter in patients with higher tumor mutational burden. Independent factors associated with shorter OS were age ≥60 years, liver metastases, low albumin levels, high LDH levels, and *KRAS* and *TP53* mutations. Outcomes for randomized patients will be reported after completion of the study.

## Introduction

In 2007, we initiated the first personalized medicine oncology program across tumor types, *I*nitiative for *M*olecular *P*rofiling and *A*dvanced *C*ancer *T*herapy (IMPACT), to explore whether in patients with advanced metastatic cancer, the use of investigational agents matched with patient tumor molecular alterations would improve treatment outcomes compared to investigational agents not matched with patient tumor alterations. In three separate cohorts of patients with advanced cancer treated from 2007 to 2013 in our Phase I Program, the overall response rates (ORRs) in the matched targeted therapy (MTT) groups ranged from 11 to 27% compared to 5% in patients treated with non-matched therapy (NMT). The median progression-free survival (PFS) or time to treatment failure ranged from 3.4 to 5.2 months in the MTT groups and from 2.2 to 2.9 months in the NMT groups, and the median overall survival (OS) ranged from 8.4 to 13.4 months in the MTT and from 7.3 to 9 months in the NMT groups^1–3^. Thus, our collective experience with the personalized medicine approach was encouraging. Of 3487 patients who underwent molecular testing, 711 received MTT and 596 received NMT. The respective ORRs were 16.4 and 5.4%; the ORR plus stable disease ≥ 6 months rates were 35.3 and 20.3%; the median PFS durations were 4.0 and 2.8 months; and the median OS durations were 9.3 and 7.3 months. As this was the first large precision medicine study across tumor types, it has the longest follow up. The 10-year OS rates were 6% vs. 1%, respectively, for the MTT and NMT groups (HR = 0.72; *p* < 0.001), and matched targeted therapy was an independent factor predicting longer OS^4^.

To overcome the limitations of IMPACT associated with the retrospective analysis of outcomes of patients who were prospectively profiled, the small number of alterations tested, and the subjective treatment assignment (selected by the treating physician), we initiated IMPACT2, a prospective randomized study in personalized medicine. The primary objective of the study is to determine whether patients treated with a matched targeted therapy selected on the basis of genomic alteration analysis of the tumor have longer PFS from the time of randomization than those whose treatment is not selected on the basis of alteration analysis. In this preliminary analysis, we describe the results of molecular profiling of 320 patients who participated in the first part of the study and assessed the association between OS and patient characteristics and molecular alterations. This analysis provides insights that have implications for the development of cancer genome-based medicine.

## Results

### Patients

**Fig. 1:**
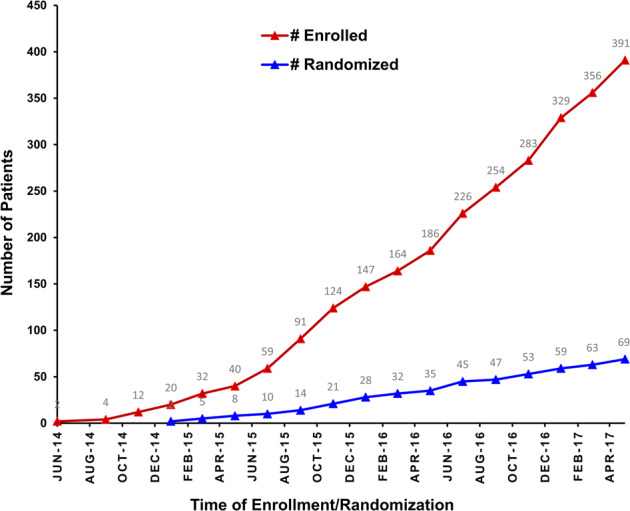
Patient enrollment and randomization by time.

From May 2014 to April 2017, 391 patients were enrolled in the first part of the study, and 320 patients (81.84%) had detectable molecular abnormalities in their tumors. Seven patients had no abnormalities on tumor molecular testing. The remaining patients had inadequate tumor cells for analysis (*n* = 19), had disease that was non-measurable/biopsy that was not feasible (*n* = 15), withdrew consent (*n* = 12), had worsening performance status (PS) (*n* = 8), had no evidence of cancer (*n* = 2), were lost to follow-up (*n* = 2), had two tumor types (*n* = 1), or were ineligible for logistic reasons (*n* = 5). Overall, 69 of the 320 patients were randomized (Fig. 1).

### Demographics

The baseline clinical characteristics of the 320 patients are shown in Table 1. The median age was 63 years (range, 25–83 years). Fifty-three percent were women. Twelve percent of patients had PS 0 and 88% had PS 1. The median time from diagnosis to enrollment on the study was 25.6 months (95% CI, 21.8–29.8 months). The median number of prior therapies was three (range, 0–14). Overall, 95% of patients had received standard therapy. Forty percent of patients had liver metastases; 24% had elevated lactate dehydrogenase (LDH) levels (≥618 IU/L, the upper limit of normal [ULN]); 15% had abnormal (above or below the normal limits) platelet counts; and 8% had low albumin levels (<lower limit of normal [LLN]). The most common cancer types were head and neck, 19%; gastrointestinal, 16%; lung, 11%; gynecological, 9%; and colorectal, 9%.

**Table 1:** Baseline characteristics.

Characteristic	Group	*N* = 320	%
Age, years	<60	128	40.0
	≥60	192	60.0
Sex	Female	170	53.1
	Male	150	46.9
No. of prior therapies	≤3	199	62.2
	>3	121	37.8
Performance status	0	40	12.5
	1	280	87.5
Platelet count, x 10^9^/L	<140	37	11.6
	140–440	271	84.7
	>440	12	3.8
No. of metastatic sites	0–2	190	59.4
	>2	130	40.6
Liver metastases	No	192	60.0
	Yes	128	40.0
LDH, IU/L	≤ULN (618)	226	70.6
	>ULN	77	24.1
	UNK	17	5.3
Albumin, g/dL	<3.5	26	8.1
	≥3.5	294	91.9
Tumor type	Head and neck	60	18.8
	Gastrointestinal, other	52	16.3
	Pancreas	18	5.63
	Esophagus	14	4.38
	GEJ	5	1.56
	Liver	5	1.56
	Stomach	5	1.56
	Small intestine	4	1.25
	Bile duct	1	0.31
	Lung	34	10.6
	Colorectal	29	9.1
	Gynecological, other	30	9.4
	Breast	26	8.1
	Sarcoma	24	7.5
	Ovarian	16	5.0
	Genitourinary, other	14	4.4
	Prostate	11	3.4
	Endocrine	9	2.8
	Other	7	2.2
	Bladder	4	1.3
	CUP	4	1.3

Abbreviations: *CUP* cancer of unknown primary, *GEJ* gastro-esophageal junction, *LDH* lactate dehydrogenase, *No.* number, *ULN* upper limit of normal.

Of 320 patients, 276 (86.25%) had molecular testing results using formalin-fixed, paraffin-embedded (FFPE) specimens derived from fresh tumor biopsies. The sites of tumor biopsy are summarized in Supplementary Table 1. For 44 (13.75%) patients, archival tumor tissue, obtained <2 years before enrollment on the study, was used. Of 276 patients, 22 patients had a second biopsy because tumor tissue was inadequate for analysis. The median time from enrollment to tumor biopsy was 8 days (range, 0–87 days); from tumor biopsy/shipment to Foundation Medicine to time of results, 20 days (range, 9–59 days); and from availability of results to initiation of treatment, 11 days (range, 5–52 days). The median time from enrollment to initiation of treatment was 2.3 months (95% CI, 1.9–2.9 months).

### Molecular testing

The hotspot mutations are summarized in Supplementary Fig. 1. The median number of variants per tumor sample was three (range, 0–31). The most common variants were missense mutations, *n* = 741; amplifications, *n* = 138; nonsense mutations, *n* = 101; and copy losses, *n* = 83. The most common variant types were single nucleotide variants (SNVs), 74%; copy gains, 17%; and copy losses, 7%. In total, 245 genes were found to be mutated in at least one patient, and the most frequently mutated genes were *TP53*, 42%; *KRAS*,16%; *PIK3CA*,12%; and *CDKN2A*, 11%.

**Fig. 2:**
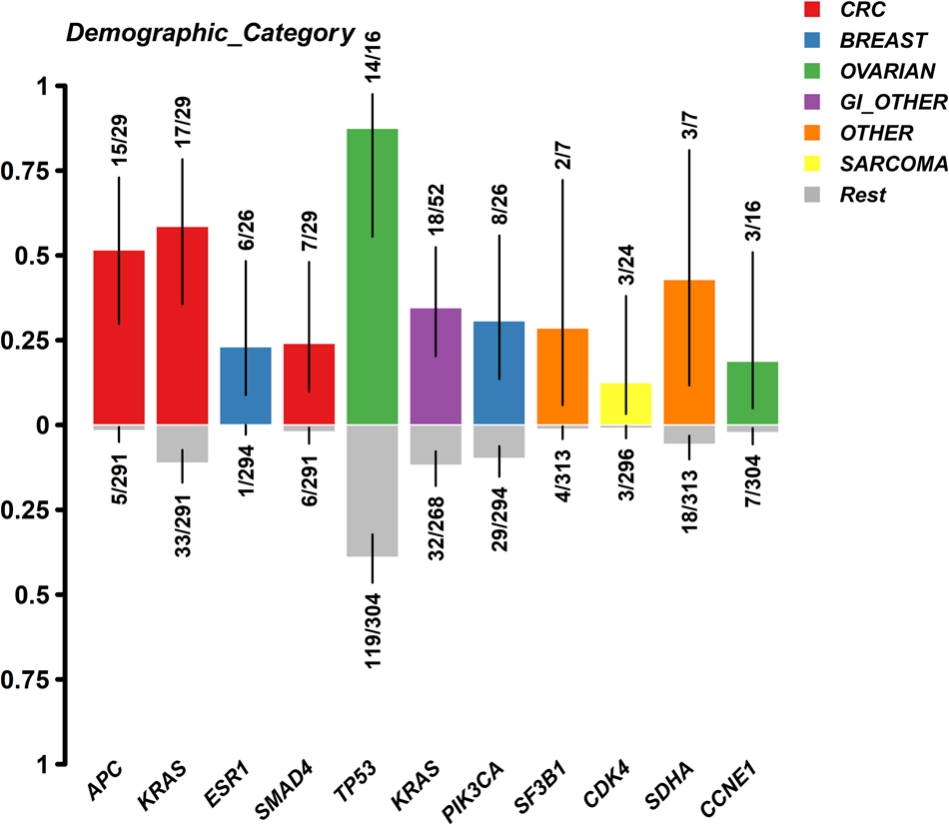
Enrichment of hotspot mutations per tumor type.

Next, we investigated the enriched abnormalities, including hotspot mutations and SNVs, according to the primary tumor types (Fig. 2). The proportion of patients with mutations in certain genes was calculated within specific tumor types and compared to the remaining tumor types. Alterations that were significantly enriched included *APC*, *KRAS*, and *SMAD4* in patients with colorectal cancer (CRC); *TP53* and *CCNE1* in patients with ovarian cancer; *PIK3CA* and *ESR1* mutations in patients with breast cancer; and *CDK4* mutations in patients with sarcoma.

**Fig. 3:**
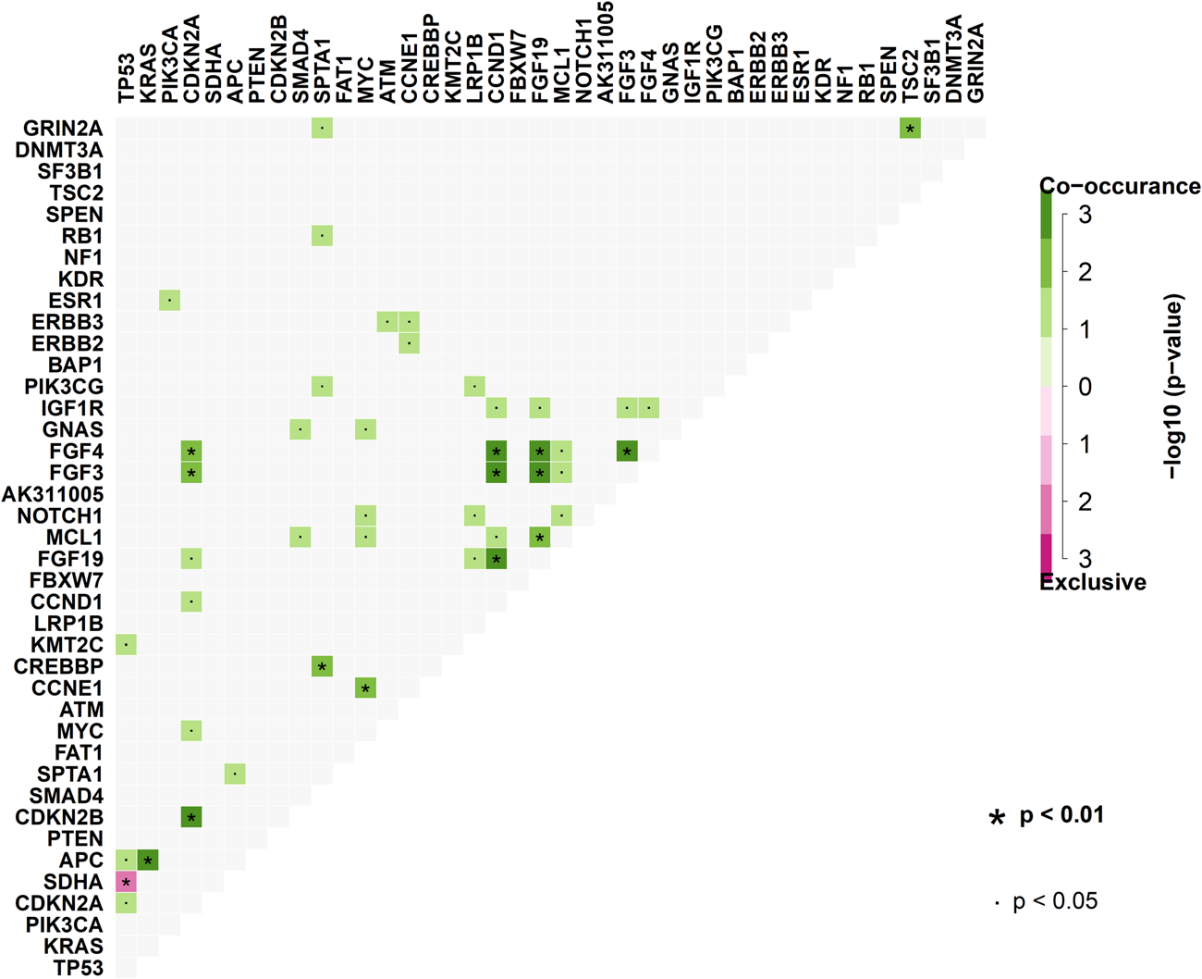
Interaction of hotspot mutations.

Statistically significant mutation interactions, representing the co-occurrence or exclusiveness of all the detected hotspot mutations, are shown in Fig. 3. For example, *APC* and *KRAS* showed strong co-occurrence. Additionally, *FGF4* and *FGF3* tended to co-occur with *CCND1*, *FGF19*, and *CDKN2A* across all patients. Notably, *TP53* showed strong mutual exclusiveness with *SDHA*.

### Treatment

Of the 69 patients who met the criteria for randomization, 60 patients received treatment and nine patients were not treated because their insurance would not cover the cost of the assigned treatment. Of the 251 patients who were not randomized, 153 (61%) were treated with investigational (*n* = 98; 64%) or standard (*n* = 55; 36%) therapy. Overall, 213 (66.56%) of the 320 patients with detectable molecular abnormalities received anticancer therapy. Fifty-six patients received treatment with immuno-oncology therapy (IO) and 157 received treatment that excluded immuno-oncology therapy (non-IO).

### Overall survival

Of the 320 patients, 202 had died at the time of the survival analysis. The median OS duration was 10.9 months (95% C.I., 8.8–12.9) and the mean OS duration was 17.1 months (95% C.I., 15.1–19.1) (Supplementary Fig. 2). Results of univariate and multivariate analyses for OS are shown in Table 2. In the univariate analysis, factors that showed significant association with shorter OS were hepatic metastases, low (<LLN) albumin level, elevated (>ULN) LDH level, older age, *KRAS* mutations, *TP53* mutations, *CDKN2A* mutations, P53 pathway abnormalities, PDGF signaling pathway abnormalities, apoptosis signaling pathway abnormalities, Ras pathway abnormalities, and T-cell activation pathway abnormalities. The following factors were not statistically significant in the univariate analyses: sex (*p* = 0.069), performance status (0 vs. 1; *p* = 0.095), number of prior therapies (continuous variable, *p* = 0.52; 0–3 vs. ≥4 lines of therapy, *p* = 0.15; Supplementary Fig. 3), number of metastatic sites (continuous variable, *p* = 0.15), platelet counts (continuous variable, *p* = 0.49), FGF/FGFR amplifications (*p* = 0.591), and tumor mutational burden (TMB) (continuous variable, *p* = 0.85). Longer time from diagnosis to enrollment on the study was associated with poorer OS (*p* < 0.0001) (Supplementary Fig. 4).

**Table 2:** Univariate and multivariate analysis for overall survival.

Univariate analysis			
Risk factor	HR	95.0% CI	*P*-value
Age ≥60 years	1.02	1.00–1.03	0.001
Liver metastases	1.70	1.28–2.24	<0.0001
LDH > 618 IU/L (ULN)	2.27	1.6–3.05	<0.0001
Albumin <3.5 g/dL (LLN)	2.00	1.33–2.99	0.001
*KRAS* mutations	2.10	1.47–3.02	<0.0001
*TP53* mutations	1.61	1.22–2.13	0.001
*CDKN2A* mutations	1.68	1.12–2.52	0.01
*P53* pathway abnormalities	1.79	1.32–2.42	<0.0001
PDGF signaling pathway abnormalities	1.35	1.00–1.83	0.049
Apoptosis signaling pathway abnormalities	1.61	1.21–2.14	0.001
Ras pathway abnormalities	1.83	1.37–2.44	<0.0001
T-cell activation pathway abnormalities	1.42	1.02–1.97	0.04
Multivariate analysis, OS			
LDH > ULN (618 IU/L)	2.19	1.61–2.97	<0.0001
*KRAS* mutations	2.27	1.57–3.28	<0.0001
Albumin < LLN (3.5 g/dL)	1.90	1.26–2.87	0.002
Age ≥60 years	1.02	1.00–1.03	0.009
Liver metastases	1.43	1.07–1.91	0.02
*TP53* mutations	1.38	1.04–1.84	0.025

Abbreviations: *LDH* lactate dehydrogenase, *LLN* lower limit of normal, *OS* overall survival, *ULN* upper limit of normal.

**Fig. 4:**
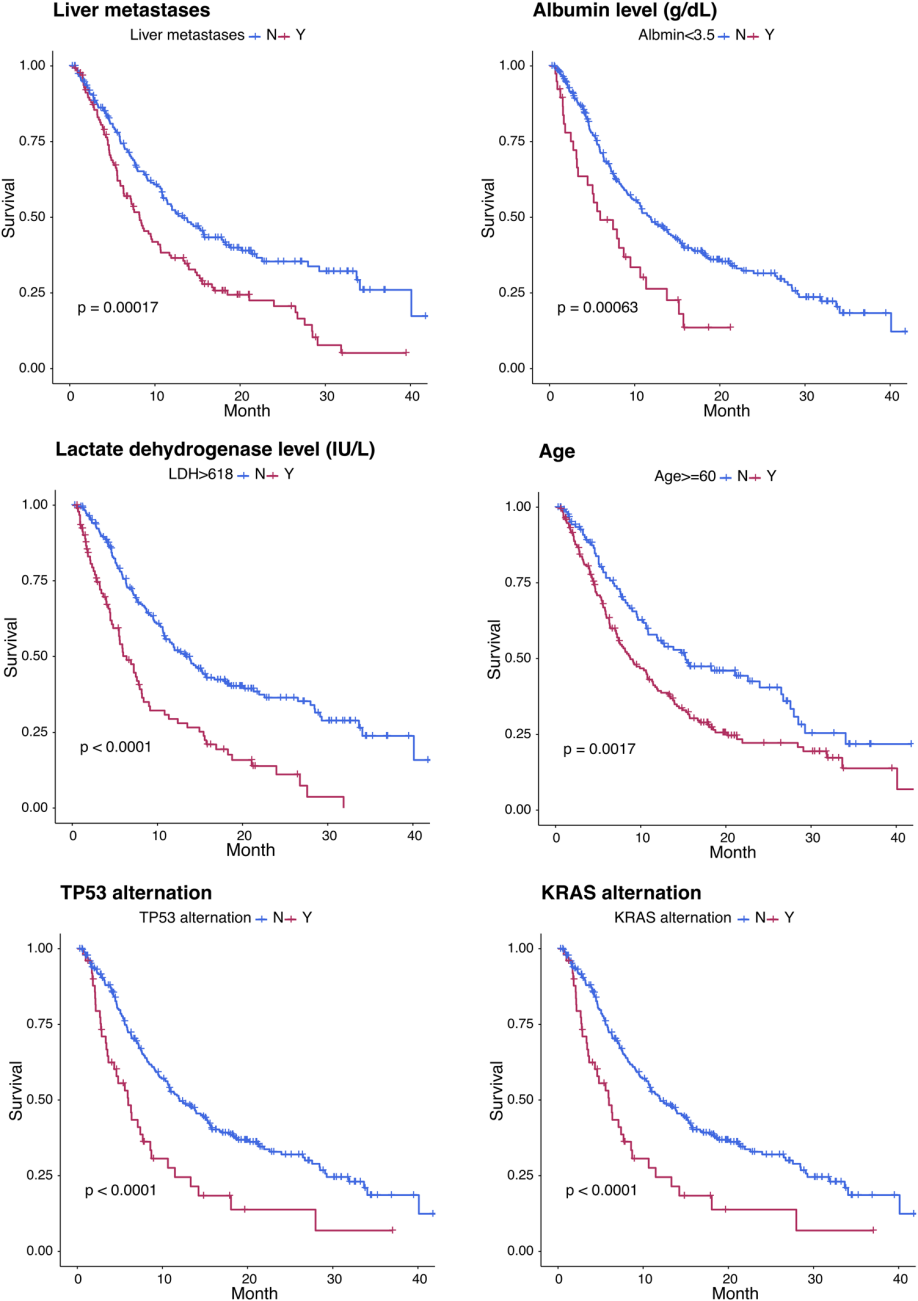
Independent risk factors predicting overall survival (multivariate analysis).

In the multivariate analysis, independent factors associated with shorter OS were age ≥60 years, liver metastases, low (<LLN) albumin levels, high (>ULN) LDH levels, and *KRAS* and *TP53* mutations (Fig. 4 and Supplementary Table 2).

The association between patients’ OS and TMB is shown in Supplementary Fig. 5. The median OS durations for patients in the high, intermediate, and low TMB groups were 7.69, 10.68, and 15.25, respectively. The low TMB group had longer OS compared to the others (*p* = 0.008, hazard ratio = 0.59). There was no significant difference in OS between patients in the high TMB and intermediate TMB groups (*p* = 0.30; hazard ratio = 1.21). We also performed OS analysis in patients with head and neck cancer by TMB. The median OS by TMB group was as follows: high, 7.23 months (95% CI, 1.18–NA); intermediate, 11.90 months (95% CI, 8.22–NA); low, not reached (95% CI, 9.07–NA) (Supplementary Fig. 6). In the remaining patients, the median OS by TMB group was as follows: high, 8.58 months (95% CI, 5.88–14.50); intermediate, 9.67 months (95% CI, 7.79–13.22); low, 13.78 months (95% CI, 6.38–29.29) (Supplementary Fig. 7).

Since “head and neck” cancers and “gastrointestinal, other” cancers were the most common tumor types, we evaluated the distribution of TMB across all tumor types (Supplementary Fig. 8). Although “head and neck” and “gastrointestinal, other” represent the tumors with the highest numbers of patients, there was no statistical difference in the distribution of the TMB groups between tumor types (“head and neck” vs. remaining tumors, “gastrointestinal, other” vs. remaining tumors, or “head and neck” + “gastrointestinal, other” vs. remaining tumors).

OS by tumor type is shown in Supplementary Table 3 and Supplementary Fig. 9. The longest OS was observed in head and neck cancer (median, 21.1 months; 95% C.I., 8.5–33.6), followed by breast cancer (median, 18.8 months; 95% C.I., 5.7–31.8), sarcoma (median, 13.8 months; 95% C.I., 6.7–21.0), and lung cancer (median, 13.5 months; 95% C.I., 7.0–20.1). Patients with cancer of unknown primary had the shortest OS (median, 1.5 months; 95% C.I., 0.0–3.7).

There was a trend towards longer OS in patients treated with IO-containing therapy compared to those treated with non-IO containing therapy (*p* = 0.069; HR = 0.68). The median OS of patients who received IO-containing therapy was 21.9 months (95% CI, 13.5–33.7 months), and the median OS of patients who received non-IO treatment was 13.3 months (95% CI, 10.8–15.8 months) (Supplementary Fig. 10).

## Discussion

IMPACT2 was initiated after the first IMPACT study demonstrated superior response, PFS, and OS for matched targeted therapy compared with unmatched therapy in consecutive patients who were referred for phase I clinical trials and had tumor molecular profiling. The aim of IMPACT2 is to assess the personalized medicine approach in a randomized study across tumor types using adaptive design to overcome the limitations of IMPACT. The execution of the trial is arduous and includes tumor biopsies for molecular testing, annotation of the genomic results, and availability of multiple clinical trials with matched and unmatched treatments. Other essential elements of IMPACT2 are randomization based on patient status on the date of clinic visit; real-time patient monitoring; efficient communication between patients, sponsors, and investigators; and accurate data collection and assessment of patient outcomes.

In this interim analysis, next-generation sequencing (NGS) testing using FFPE specimens from fresh tumor biopsies and treatment of patients with advanced metastatic cancer prospectively was feasible. Overall, 81.84% of the enrolled patients had detectable molecular abnormalities, and 66.56% of 320 patients received anticancer therapy. The median OS duration was 10.9 months (95% C.I., 8.8–12.9) and the mean OS duration was 17.1 months (95% C.I., 15.1–19.1). These survival data are compatible with previous reports on patients who were treated in our Phase I Program^1–5^.

The relatively small proportion (21.6%) of patients who met criteria for randomization was attributed to lack of clinical trials with targeted therapies against genetic alterations in individual patients; worsening performance status; ineligibility for clinical trials with targeted therapy, particularly due to comorbidities; and logistics issues (drugs were not provided at no cost to patients, as in the NCI MATCH or American Society of Clinical Oncology TAPUR [Targeted Agent and Profiling Utilization Registry] studies).

The median number of variants per tumor type (3, range, 1–31) and the distribution of the alterations (741 missense mutations, 138 amplifications, 101 nonsense mutations, and 83 copy losses) reflect the advanced stage and complexity of the patients’ tumors. Most alterations were SNVs (74%) followed by copy gains (17%) and copy losses (7%), with C > T being the most frequent base substitution. The large number of genes (*n* = 245) that were mutated in ≥1 patient emphasizes the need to develop effective targeted agents against each gene that may drive carcinogenesis in humans. The distribution of the most frequently mutated genes (*TP53*, 42%; *KRAS*, 16%; *PIK3CA*, 12%; and *CDKN2A*, 11%) is consistent with that in our published data in patients with advanced cancer referred for investigational therapy.

Findings of the enrichment analysis of hotspot mutations and CNVs per tumor type are in line with published data (*APC*, *KRAS*, and *SMAD4* mutations in CRC; *TP53* and *CCNE1* in ovarian cancer; *PIK3CA* and *ESR1* in breast cancer; and *CDK4* in sarcoma [Fig. 2]). The co-occurrence of mutations may be associated with their presence in specific tumor types, i.e., the *APC* and *KRAS* alterations (*p* < 0.01) in CRC. A trend towards co-occurrence of *FGF4* and *FGF3* with *CCND1*, *FGF19*, and *CDKN2A* was also noted across all patients. The strong mutual exclusiveness (*p* < 0.01) of *TP53* hotspot mutations (42% of 320 patients) and *SDHA* hotspot mutations (7% of 320 patients) may provide insights regarding the role of these genes in carcinogenesis (Fig. 3).

The duration of OS decreased as TMB increased (median: 15.25 months with low TMB; 10.68 months with intermediate; and 7.69 months with high; Supplementary Fig. 5). The longer OS of patients with low TMB compared to those with intermediate or high TMB is likely attributable to their relatively favorable tumor biology owing to fewer molecular abnormalities. Biomarkers for selection of immunotherapy and immuno-oncology clinical trials were limited at the time of initiation of IMPACT2, and therefore, very few patients received immunotherapy.

Whereas we used the top and bottom 20% of TMB values to define high and low TMB, respectively, other investigators have used different cut-off values^6–11^. High TMB has been defined as >23.1 mutations/Mb in patients with melanoma^9^; >100 mutations per tumor in patients with melanoma treated with antibodies against cytotoxic T-lymphocyte antigen 4 (CTLA-4)^11^; >10 mutations/Mb in patients with non-small cell lung cancer^7^; and ≥ 20 mutations/Mb in patients with various tumor types treated with immuno-oncology therapy^10^. In the latter study, high TMB was associated with better clinical outcomes compared to patients with lower TMB. High TMB has been associated with response to immune checkpoint inhibitors. Although the relatively small number of patients precluded robust analyses, and patients were treated on numerous clinical trials, the OS analysis by TMB demonstrated a better separation of the survival curves in the head and neck patient group compared to others (Supplementary Figs. 6 and 7). Caution is warranted in the interpretation of these results that indicate that the individual tumor types should be taken into consideration in assessing the role of TMB in OS. Taking into consideration our findings and published results, it is plausible that the clinical significance of TMB is at least partially associated with driver molecular alterations (that may impact clinical outcomes more than TMB), tumor type, test performance, cut-off point, and/or type of therapy, including immuno-oncology therapy. Ongoing clinical trials are testing the importance of this biomarker. Standardization and consensus about the use of TMB would be useful.

Independent factors associated with shorter OS in the multivariate analysis were elevated LDH levels (*p* < 0.0001), low albumin levels (*p* = 0.002), liver metastases (*p* = 0.02), age ≥60 years (*p* = 0.009), *KRAS* mutations (*p* < 0.0001), and *P53* mutations (*p* = 0.025) (Fig. 4 and Supplementary Table 2). The first three factors indicate advanced disease and the first four are established markers associated with poorer outcomes. The association of *KRAS* and *P53* mutations with shorter OS may be explained at least in part by the essential role of these biomarkers in carcinogenesis and the lack of effective targeted therapies against these alterations.

Several other trials have investigated the role of precision medicine in treating cancer. In SHIVA, a multicenter French trial, 293 of 741 enrolled patients had ≥1 molecular alteration and were treated with one of 11 targeted therapies. No difference in PFS was noted between the two arms, although the study design was suboptimal^12,13^. The TAPUR study is evaluating U.S. Food and Drug Administration-approved treatments in patients with advanced cancer and potentially actionable molecular alterations, providing real-world data. As of April 2020, nine arms of that study were expanded, and seven arms were closed^14^. NCI-MATCH, a phase II non-randomized trial, evaluates the clinical benefit of targeted treatments matched to tumor molecular alterations in patients with refractory malignancies^15^. Although few of the enrolled patients (16 of 645) were treated in the initial analysis, the study was amended to allow patients with clinical laboratory improvement amendments (CLIA)-certified NGS testing available at study entry. Some subprotocols demonstrated encouraging results, i.e., in patients with deficient mismatch repair and advanced non-colorectal tumors treated with nivolumab, the ORR was 36% (all partial responses) and the median OS was 7.3 months^16^. In patients with tumor *AKT1* E17K mutation (0.77% frequency) treated with the pan-AKT inhibitor capivasertib, the ORR was 23% (all partial responses) and the 6-month PFS was 52%^17^. In contrast to IMPACT2, where the regulatory institutional committees required a change in the eligibility criteria from 0 to 2 prior therapies to enroll patients who have exhausted all standard options, NCI-MATCH enrolls patients with ≥1 standard systemic therapy and no other treatments available that are known to prolong OS^18^.

The strengths of IMPACT2 include the prospective nature of the study; the implementation of FFPE specimens derived from fresh tumor biopsies for NGS, which was not the standard of care when IMPACT2 was initiated; the inclusion of state-of-the-art NGS testing (315-gene panel, 27-gene amplification testing); and discussion of the clinical significance of NGS results at the study’s tumor molecular board and at a multidisciplinary conference to optimize treatment selection. The most important strength is access to a broad portfolio of cutting-edge early phase clinical trials against multiple targets offered by health care providers with expertise in drug development. The weaknesses of our trial include the variety of tumor types, the multiplicity and complexity of molecular alterations, the inherent limitations of treating advanced, metastatic cancer, and the variety of investigational agents that change over time. Although the availability and efficiency of molecularly driven studies increases over time, our study—like other clinical trials across tumor types—cannot systematically account for all differences in tumor biology and characteristics of individual patients for optimal treatment selection.

In conclusion, IMPACT2 establishes the feasibility of tumor biopsies for genomic profiling in patients with solid tumors—that was not the standard practice—and prospective treatment of patients. Tumor board and multidisciplinary review of molecular alterations and available clinical trials optimizes personalized treatment selection. In the study patient population, age <60 years, no liver metastases, normal albumin and LDH levels, and absence of *KRAS* or *TP53* mutations were independent factors predicting longer OS. Outcomes for randomized patients will be reported after completion of the study, which continues to accrue patients. Our data contribute to evolving clinical research that offers comprehensive molecular testing to help select efficacious targeted therapy to accelerate drug approval. Optimization of biomarker testing using tumor and cell-free DNA and integration of innovative targeted therapeutic approaches will advance the landscape of precision oncology, enabling delivery of personalized care to more patients with cancer.

## Methods

### Eligibility criteria

Patients were eligible if they were ≥18 years of age and had advanced or metastatic cancer that was refractory to standard-of-care therapy, had declined to receive standard-of-care therapy, or had no standard-of-care therapy available for their tumor type. The study was registered in www.clinicaltrials.gov (NCT02152254). Patients could have received unlimited lines of prior therapy. Other eligibility criteria included a European Cooperative Oncology Group PS of 0-1 and adequate bone marrow (absolute neutrophil count ≥1000/µL; platelets ≥100,000/µL), hepatic (total bilirubin level ≤1.5 times the ULN, unless the patient has known Gilbert’s disease, and alanine aminotransferase/serum glutamic pyruvic transaminase levels ≤2.5 times the ULN without liver metastases), and renal (serum creatinine clearance ≥50 mL/min by the Cockcroft-Gault formula) function. Patients with brain metastases were eligible to participate in the study if the metastases were stable (treated and asymptomatic) and the patient was off steroids for at least 2 weeks. Patients with a previous malignancy (other than the patients’ known cancer) who were treated successfully and were disease-free for at least 3 years and patients with a history of basal cell carcinoma of the skin or pre-invasive carcinoma of the cervix were not excluded from the study. Women of childbearing potential were required to use adequate contraception (hormonal or barrier method of birth control; abstinence) prior to study entry and for the duration of study participation.

Exclusion criteria included anticancer treatment within 3 weeks of initiating study treatment, ≥grade 2 adverse events associated with prior therapy, uncontrolled hypertension, angina, ventricular arrhythmias, congestive heart failure (New York Heart Association Class ≥II), prior or current cardiomyopathy, atrial fibrillation with heart rate >100 bpm, unstable ischemic heart disease, peripheral neuropathy ≥grade 2, pregnancy, concurrent severe and/or uncontrolled medical disease that could compromise participation in the study (i.e., uncontrolled diabetes, severe infection requiring active treatment, severe malnutrition, chronic severe liver or renal disease), and any other condition that would, in the investigators’ judgment, contraindicate the patient’s participation in the clinical study due to concerns about safety or compliance with clinical study procedures. For oral therapy only, patients were excluded from the study if they had gastrointestinal diseases that would preclude adequate absorption.

Although the study was optimally designed to enroll patients with 0–2 prior therapies, the initial accrual rate was too low, prompting an amendment to allow patients with unlimited lines of therapy. Therefore, the original criterion allowing patients with 0–2 prior therapies was updated according to evolving institutional guidelines to require that the patient’s cancer was refractory to standard-of-care therapy, the patient declined to receive standard-of-care therapy, or there was no standard-of-care therapy available for the patient’s tumor type. All patients signed informed consent forms stating that they were aware of the investigational nature of the study. The study adhered to the guidelines of the Institutional Review Board, which approved the study. The study was activated in May 2014.

### Tumor molecular profiling

**Fig. 5:**
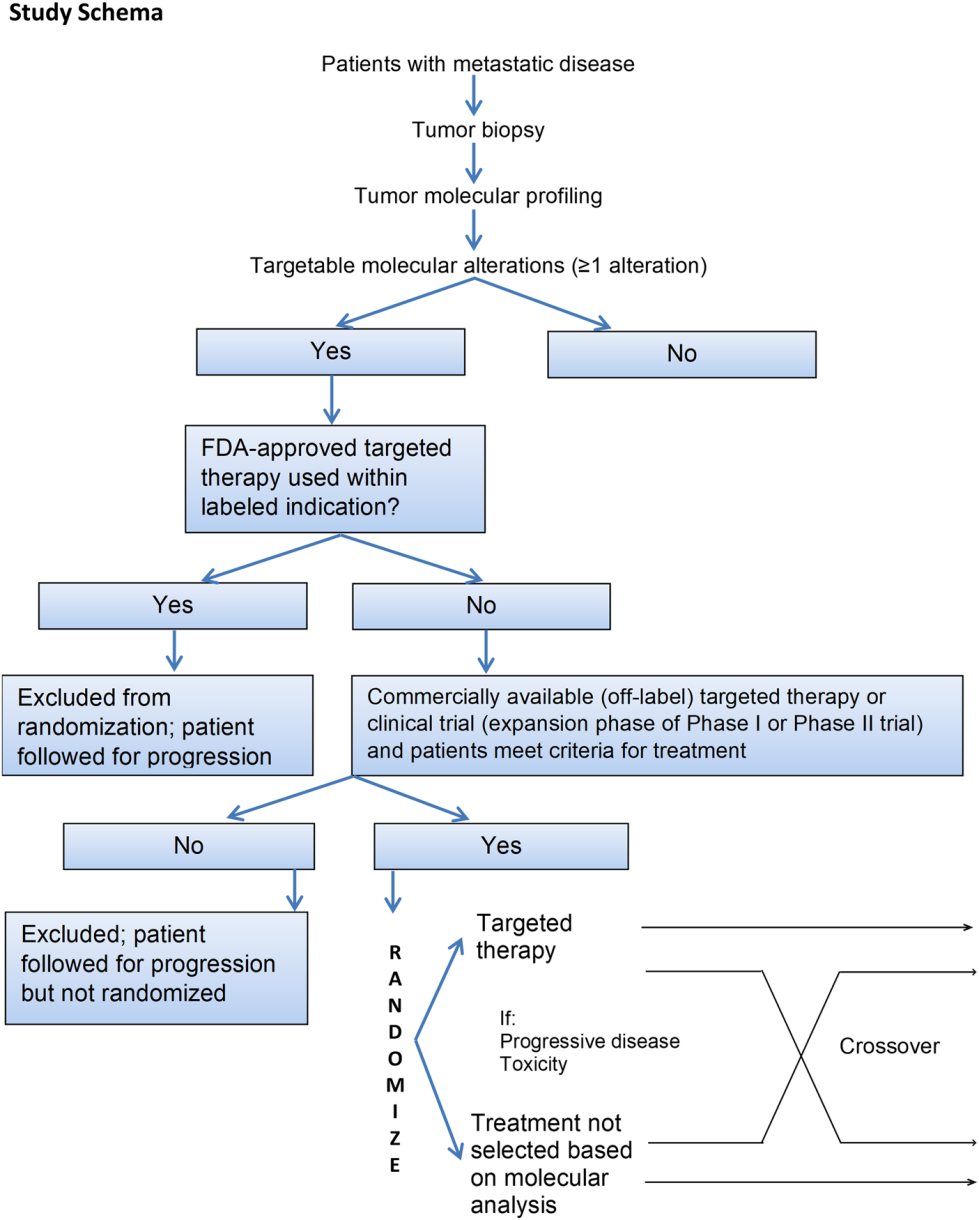
Study schema.

The study schema is shown in Fig. 5. Patients who were eligible for the study underwent tumor biopsy. Tumor samples were obtained by core biopsy performed by an interventional radiologist or bronchoscopy. FFPE specimens derived from fresh tumor biopsies were sent to Foundation Medicine for molecular profiling. It should be noted that at the sponsor’s request, the study was placed on hold in April 2017. Subsequently, patients enrolled from trial initiation until April 2017 constituted the “first part” of the study.

### Next-generation sequencing

Prior to shipment to Foundation Medicine, tumor specimens were reviewed by an MD Anderson pathologist to ensure adequate tumor cellularity (≥20%) for analysis. All procedures were performed in a CLIA-compliant environment.

Patient samples were sequenced by Foundation Medicine, Inc. (Cambridge, MA), using FoundationOne CDx™, a comprehensive NGS-based in vitro diagnostic device designed to capture cancer genes. CDx™ detects substitutions, insertion and deletion alterations (indels), and copy number alterations in 324 genes and selected gene rearrangements in DNA isolated from FFPE tumor tissue samples. Genomic DNA was extracted from 40 µm of tissue using the Promega Maxwell 16 FFPE Plus LEV DNA Purification kit (Madison, WI) according to the manufacturer’s instructions and quantified using a standardized PicoGreen fluorescence assay (Invitrogen, Carlsbad, CA). At least 50 ng and up to 200 ng of extracted DNA was sheared to ~100–400 bp by sonication before end-repair, dA addition, and ligation of indexed, Illumina-sequencing adaptors (San Diego, CA). Sequencing libraries were hybridization-captured using a pool of >24,000 custom-designed and individually synthesized 5′-biotinylated DNA oligonucleotides (Integrated DNA Technologies, Coralville, IA). These baits were designed to target ~1.5 Mb of the human genome, including 4,604 exons of 324 genes related to cancer therapy, 47 introns of 19 genes frequently re-arranged in cancer, and 3549 SNPs throughout the genome. DNA sequencing was performed using the HiSeq-2000 instrument (Illumina), with 49 × 49 paired-end reads.

### Treatment

Results of molecular testing as provided by Foundation Medicine were presented at weekly or bimonthly meetings of the study’s tumor board, which consisted of the study oncologists, statistician, radiologist, and molecular biologists. Patients were also presented at a weekly multidisciplinary conference to optimize treatment selection. Subsequently, they were seen in clinic and were randomized to receive matched targeted therapy selected on the basis of genomic alteration analysis or treatment not selected on the basis of alteration analysis. Treatment was also determined on the basis of whether the patient had ≥1 targetable alteration, clinical trials were available, the patient met eligibility criteria for the clinical trials under consideration, and insurance approved coverage of the associated cost. Stratification factors for randomization (determined in 2013) included alterations in the following genes: *KRAS*, *BRAF*, *ERBB2* (Her2), *EGFR*, *PIK3CA*, *PTEN*, *MET*, or “Other” (i.e., remaining tumor alterations). Patients who did not meet the criteria for study randomization were treated with therapies chosen by their treating physician.

### Exploratory analyses

Results, provided by Foundation Medicine at the time of completion of molecular testing, were used to guide therapy. We also performed the following exploratory analyses.

### Variant annotation and pathway analysis

A customized workflow pipeline was applied to analyze the sequencing results; the pipeline was adapted from tools that are applied to cancer genome sequencing projects such as TCGA but implemented with further optimization for deep clinical sequencing. Briefly, we aligned the reads to human reference assembly hg19 using BWA and Picard. MuTect was used to identify somatic point mutations, and Pindel was used to identify somatic insertions and deletions. To eliminate artifact calls and germline contamination, a series of post-call filtering algorithms for somatic mutations were applied: (a) total read count in tumor sample ≥20, (b) log of odds score ≥20, (c) variant allele frequency ≥0.02 in tumor sample, and (d) population frequency threshold of 0.15% for filtering out common variants in the databases dbSNP, 1000 Genome Project, Exome Aggregation Consortium, and ESP6500. Mutations were annotated using ANNOVAR and hotspot mutations were annotated using the COSMIC database. The variants that passed the filtering but were not reported in COSMIC were annotated as variants of unknown significance. An unbiased pathway enrichment analysis was performed using the Panther pathway database (http://pantherdb.org/) (Supplementary Table 4). In this analysis, we evaluated the association between patient’s OS and patient tumor alterations on both the gene level and pathway level (Table 2). As the alterations on the pathway level are highly dependent on the genes included in the panel, our goal was to select the pathways without introducing any bias. Therefore, we performed the pathway enrichment analysis on the gene panel to identify the pathways that are reflected, and then we performed the univariate analysis on the pathways. The pathways reflected by this gene panel that were included in the univariate analysis are listed in Supplementary Table 4. Table 2 shows the gene/pathway alterations that were significantly associated with patient OS.

### Tumor mutational burden analysis

Patients were divided into three groups according to the TMB identified in DNA sequencing. The TMB cut-off points were determined taking into consideration the following two criteria: (1) the burden of the high TMB group should be at least two-fold higher than that of the low TMB group; (2) the cutoff should reflect the curve of TMB distribution. Using these criteria, we set the cutoff of the top 20% (mutation load ≥18) as the high TMB group, the bottom 20% (mutation load <9) as the low TMB group, and the remaining as the intermediate TMB group. The majority of patients were in the intermediate TMB group.

### Statistical methods

*R*esponse *E*valuation *C*riteria *I*n *S*olid Tumors (RECIST) guidelines were used to evaluate tumor response and disease progression every two cycles^19,20^. OS was calculated from the date of consent to the date of death from any cause or last follow-up. Cox regression analysis was used to determine the association between OS and patient pretreatment characteristics. The risk factors that were statistically significant in the univariate analysis were further selected for the multivariate analysis, using the backward stepwise selection elimination method (likelihood ratio). According to this method, removal testing is based on the probability of the likelihood-ratio statistic, which is based on the maximum partial likelihood estimates. The cut-off time for this analysis was June 2019.

### Reporting summary

Further information on research design is available in the Nature Research Reporting Summary linked to this article.

## Supplementary information

Supplementary Information

Reporting Summary

## Data Availability

The next-generation sequencing data generated during the study, are available in the European Genome-phenome Archive (EGA) (data are subject to controlled-access): https://identifiers.org/ega.dataset:EGAD00001006887 (dataset ID) and https://identifiers.org/ega.study:EGAS00001004964 (study ID)^21^. The dataset IMPACT2_supporting_data.xlsx, supporting Fig. 4, Tables 1 and 2, Supplementary Tables 1–3 and Supplementary Figs. 2–10, is part of the supplementary files that accompany the article. For data inquiries, please contact the corresponding author Dr. Apostolia-Matia Tsimberidou, email address: atsimber@mdanderson.org. The data generated and analyzed during this study are described in the following metadata record: 10.6084/m9.figshare.13643420^22^.
