# Supplementary material for: Precision medicine: preliminary results from the Initiative for Molecular Profiling and Advanced Cancer Therapy 2 (IMPACT2) study
Source: NPJ Precis Oncol. 2021 Mar 19;5:21. doi: 10.1038/s41698-021-00159-2 (PMC7979841; doi:10.1038/s41698-021-00159-2)
Supplement: Supplementary file 1 — Supplementary Information [file 41698_2021_159_MOESM1_ESM.docx]

**SUPPLEMENTAL INFORMATION**

**Supplementary Table 1.** Sites of tumor biopsy

| **Location** | ***N=* 320** | **%** |
| --- | --- | --- |
| Liver | 90 | 28.13 |
| Lung | 59 | 18.44 |
| Lymph node | 38 | 11.88 |
| Abdominal/peritoneum | 33 | 10.63 |
| Neck/ear/larynx/oral & nasal cavity | 20 | 6.25 |
| Soft tissue | 18 | 5.63 |
| Chest wall/pleura | 18 | 5.63 |
| Pelvis | 8 | 2.5 |
| Bone | 8 | 2.5 |
| Adrenal gland | 6 | 1.88 |
| Retroperitoneum | 7 | 1.88 |
| Breast | 4 | 1.25 |
| Kidney | 2 | 0.63 |
| Skin | 2 | 0.63 |

The remaining patients had a tumor biopsy in the following organs (1 each): colon, mediastinum, ovary, pubic mass, prostate, spine, stomach.

**Supplementary Table 2.** Independent risk factors predicting overall survival (multivariate analysis)

| **Risk Factor** | | **Mean** | | | **Median** | | |
| --- | --- | --- | --- | --- | --- | --- | --- |
|  |  | **Estimate** | **Std. Error** | **95% CI** | **Estimate** | **Std. Error** | **95% CI** |
| **Liver metastases** | No (*N*=192) | 20.15 | 1.43 | 17.35-22.95 | 13.22 | 1.64 | 10.02-16.43 |
|  | Yes (*N*=128) | 12.63 | 1.07 | 10.53-14.72 | 8.19 | 0.88 | 6.47-9.92 |
| **Albumin < 3.5 g/dL** | No (*N*=294) | 17.69 | 1.05 | 15.63-19.75 | 11.47 | 1.25 | 9.03-13.92 |
|  | Yes (*N*=26) | 7.48 | 1.23 | 5.06-9.90 | 7.46 | 2.80 | 1.98-12.94 |
| **LDH > 618 IU/L** | No (*N*=226) | 19.95 | 1.26 | 17.48-22.42 | 13.32 | 1.30 | 10.77-15.87 |
|  | Yes (*N*=94) | 9.70 | 1.03 | 7.68-11.72 | 6.38 | 0.88 | 4.66-8.10 |
| **Age ≥ 60 yrs** | No (*N*=128) | 20.00 | 1.53 | 17.00-23.00 | 15.25 | 3.83 | 7.74-22.76 |
|  | Yes (*N*=192) | 14.79 | 1.18 | 12.48-17.10 | 8.78 | 1.19 | 6.44-11.12 |
| ***TP53* mutated** | No (*N*=187) | 20.13 | 1.44 | 17.30-22.96 | 12.76 | 2.20 | 8.45-17.07 |
|  | Yes (*N*=133) | 13.20 | 1.17 | 10.91-15.48 | 8.58 | 1.28 | 6.08-11.08 |
| ***KRAS* mutated** | No (*N*=270) | 18.28 | 1.11 | 16.11-20.45 | 12.00 | 1.24 | 9.57-14.44 |
|  | Yes (*N*=50) | 9.78 | 1.68 | 6.49-13.07 | 5.98 | 0.97 | 4.07-7.89 |

**Supplementary Table 3.** Overall survival by tumor type

| **Tumor type** | **1-year OS, %** | **Mean** | **Median, months** |
| --- | --- | --- | --- |
| Bladder | 25.0 | 9.08 | 7.4 |
| Breast | 66.0 | 22.54 | 18.77 |
| Colorectal | 38.4 | 11.16 | 7.69 |
| Endocrine | 66.7 | 24.35 | Not reached |
| Gastrointestinal, other | 23.6 | 8.87 | 5.92 |
| Genitourinary, other | 50.0 | 20.32 | 12 |
| Gynecological, other | 47.6 | 14.99 | 12 |
| Head and neck | 52.3 | 21.46 | 21.07 |
| Lung | 53.7 | 15.13 | 13.51 |
| Ovarian | 54.2 | 17.1 | 12.53 |
| Prostate | 40.4 | 11.38 | 11.7 |
| Sarcoma | 51.5 | 20.19 | 13.84 |
| Unknown primary | Not reached | 2.89 | 1.51 |
| Other | 42.9 | 10.88 | 10.62 |

**Supplementary Table 4.** Enriched pathways

**SUPPLEMENTARY FIGURE LEGENDS**

**Supplementary Figure 1.** Summary of somatic hotspot mutations

**Supplementary Figure 2.** Overall survival of 320 patients

**Supplementary Figure 3.** Overall survival by number of prior therapies

**Supplementary Figure 4.** Overall survival by time from diagnosis to enrollment on the study

**Supplementary Figure 5.** Overall survival by tumor mutational burden

**Supplementary Figure 6.** Overall survival by tumor mutational burden in patients with head and neck cancer

**Supplementary Figure 7.** Overall survival by tumor mutational burden in patients with cancer type other than head and neck cancer

**Supplementary Figure 8.** Distribution of tumor mutational burden group by tumor type. The proportion of patients with low TMB was 21.6% in head and neck (H&N), 17.3% in the remaining tumor types excluding gastrointestinal, other (GI_O) (p=0.45), and 16.9% in all remaining tumor types, including GI_O (p=0.45). The proportion of patients with high TMB was 13.3% in H&N, 20.7% in others tumor types excluding GI_O (p=0.26), and 20% in all remaining tumor types, including GI_O (p=0.27). Similarly, the proportion of patients with low TMB was 15.4% in GI_O, 17.3% in others tumor types excluding H&N (p=0.84), and 18.3% in all other tumor types including H&N (p=0.70). The proportion of patients with high TMB was 17.3% in GI_O, 20.7% in other tumor types excluding H&N (p=0.70), and 19% in all other tumor types including H&N (p=0.85). All comparisons were performed using the Fisher exact test. Abbreviations: BLCA: Bladder; BRCA: Breast; CRC: Colorectal; ENDO: Endocrine; GI_O: Gastrointestinal, other; GU_O: Genitourinary, other; GYN_O: Gynecological, other; H&N: Head and neck; LUNG: Lung; OVA: Ovarian; PRO: Prostate; SAR: Sarcoma; UNKNOWN: Unknown primary.

**Supplementary Figure 9.** Overall survival time by tumor type. Abbreviations: BLCA: Bladder; BRCA: Breast; CRC: Colorectal; ENDO: Endocrine; GI_O: Gastrointestinal, other; GU_O: Genitourinary, other; GYN_O: Gynecological, other; H&N: Head and neck; LUNG: Lung; OVA: Ovarian; PRO: Prostate; SAR: Sarcoma; UNKNOWN: Unknown primary.

**Supplementary Figure 10**. Overall survival by type of therapy (immunotherapy vs non-immunotherapy)

**SUPPLEMENTARY FIGURES**

**Supplementary Figure 1**

**
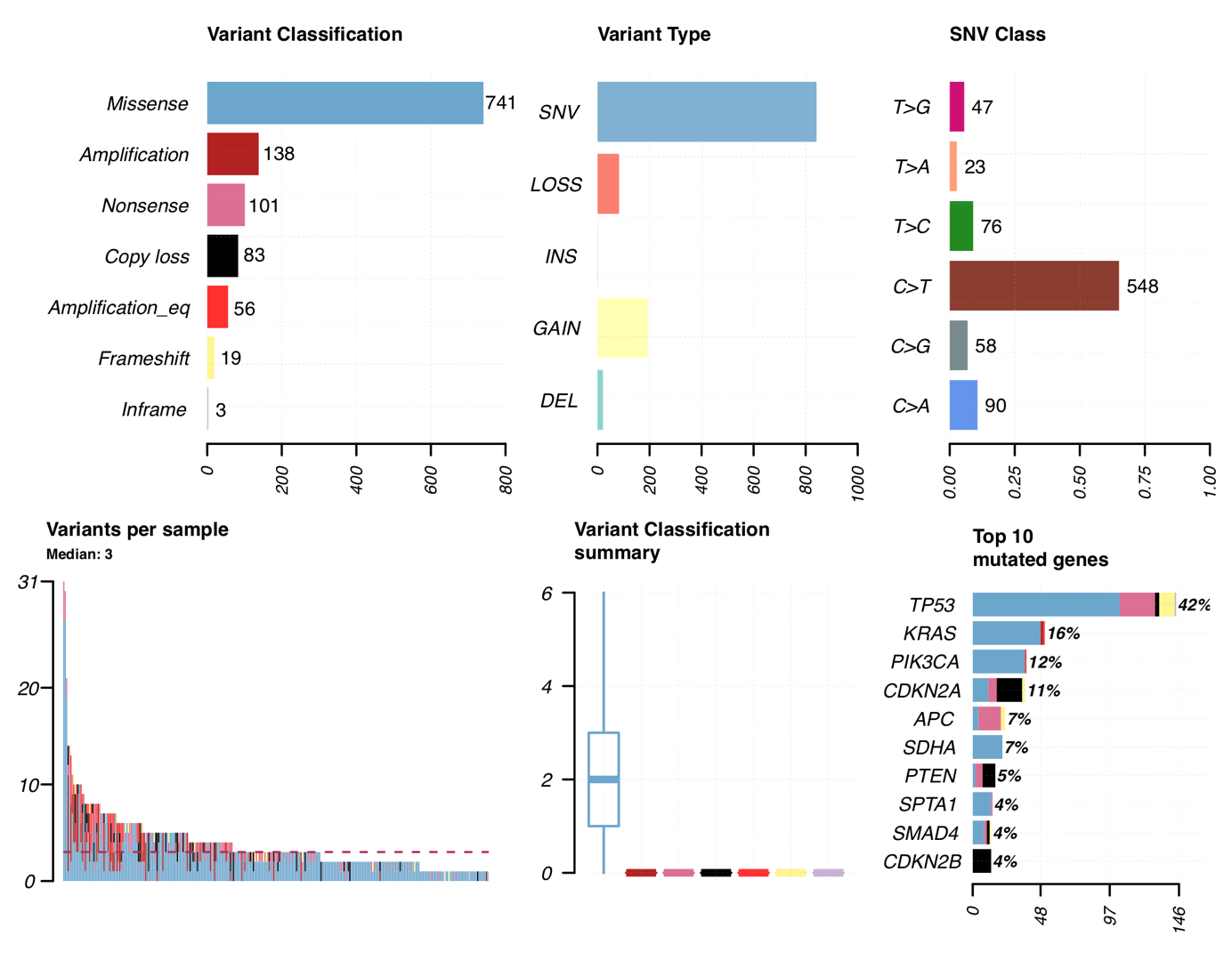
**

**Supplementary Figure 2**

**
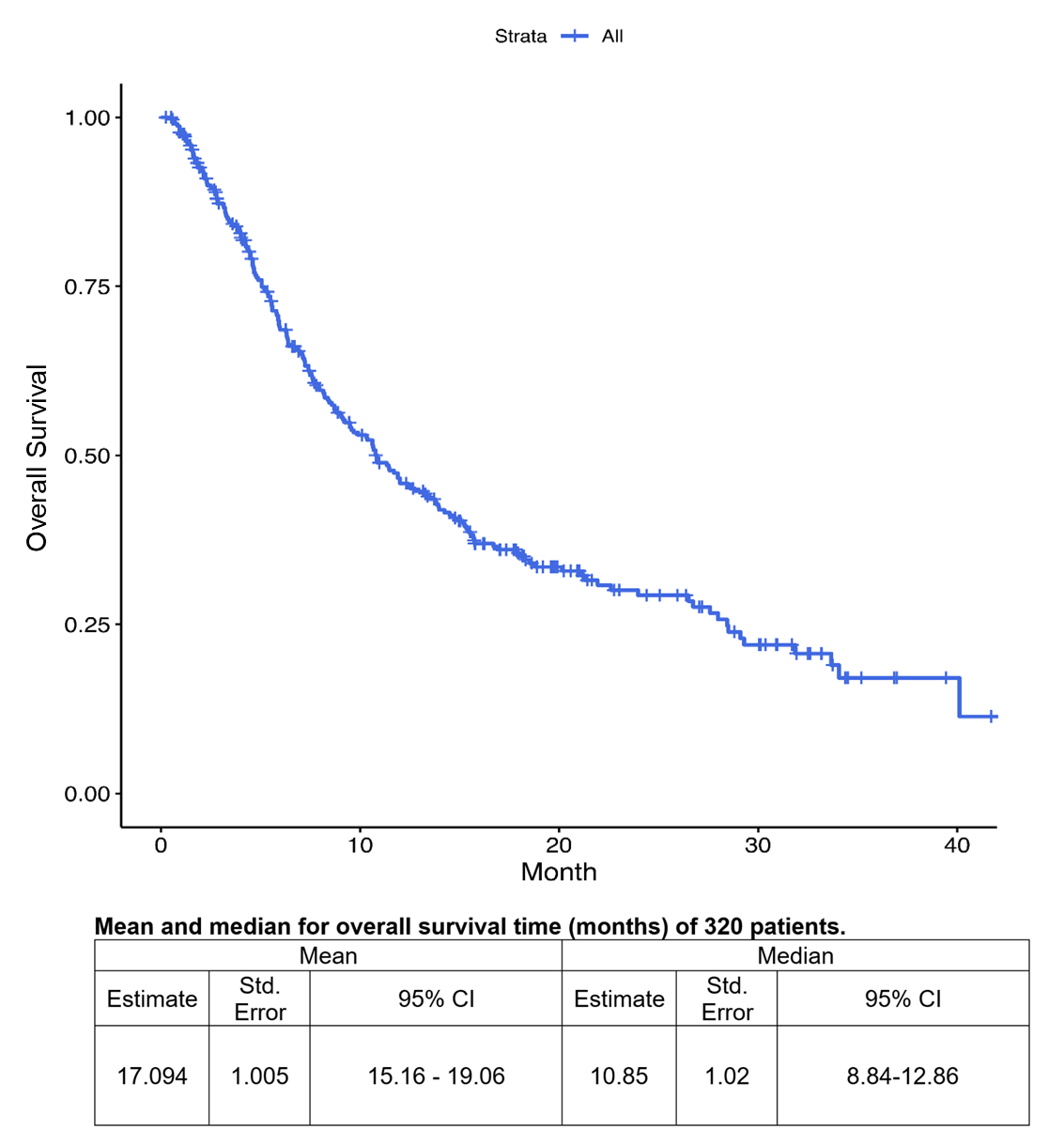
**

**Supplementary Figure 3**


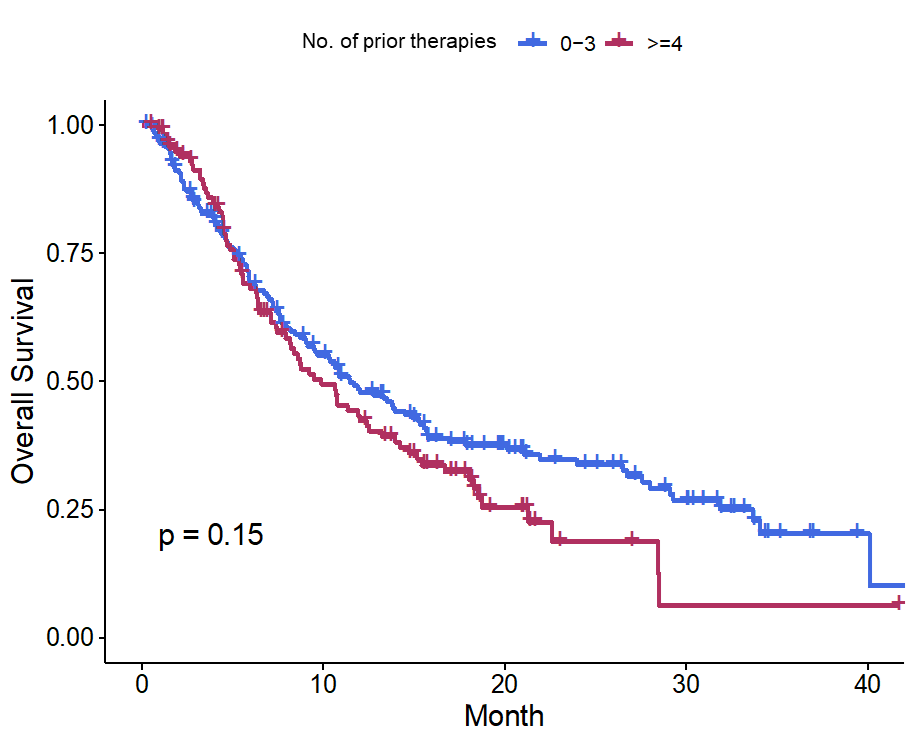


**Supplementary Figure 4**

**Supplementary Figure 5**

**
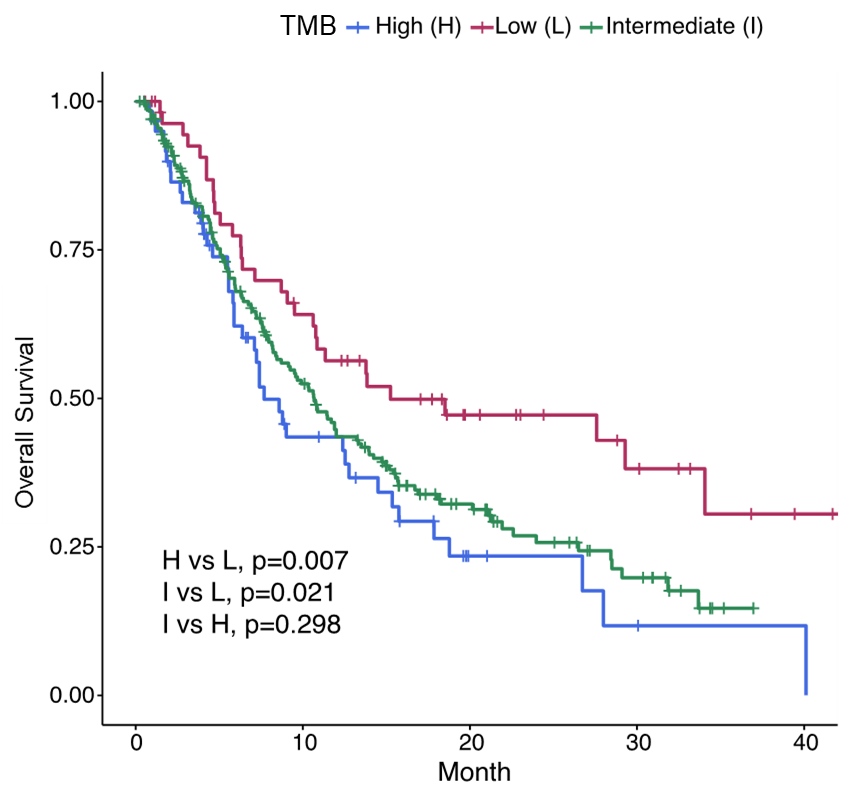
**

| Mutation load | Mean | | | Median | | |
| --- | --- | --- | --- | --- | --- | --- |
|  | Estimate | Std. Error | 95% CI | Estimate | Std. Error | 95% CI |
| Low (N=57) | 23.23 | 2.60 | 18.137-28.31 | 15.25 | 8.48 | 0-31.88 |
| Intermediate (N=203) | 15.27 | 0.98 | 13.352-17.19 | 10.68 | 0.97 | 8.775-12.59 |
| High (N=60) | 13.75 | 1.90 | 10.027-17.48 | 7.69 | 0.98 | 5.763-9.62 |

**Supplementary Figure 6**

**
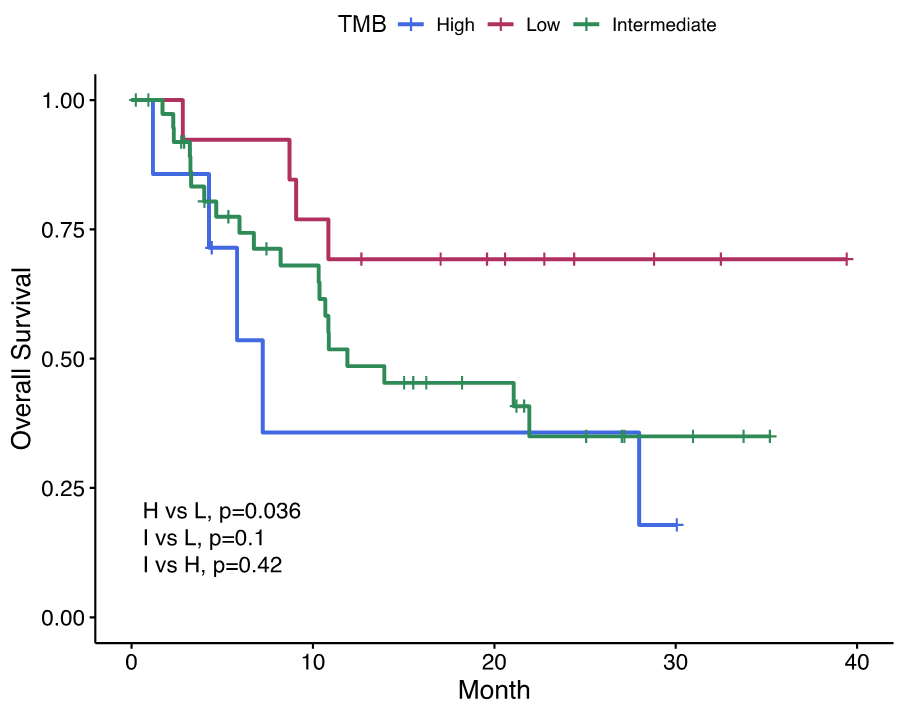
**

**Supplementary Figure 7**

**
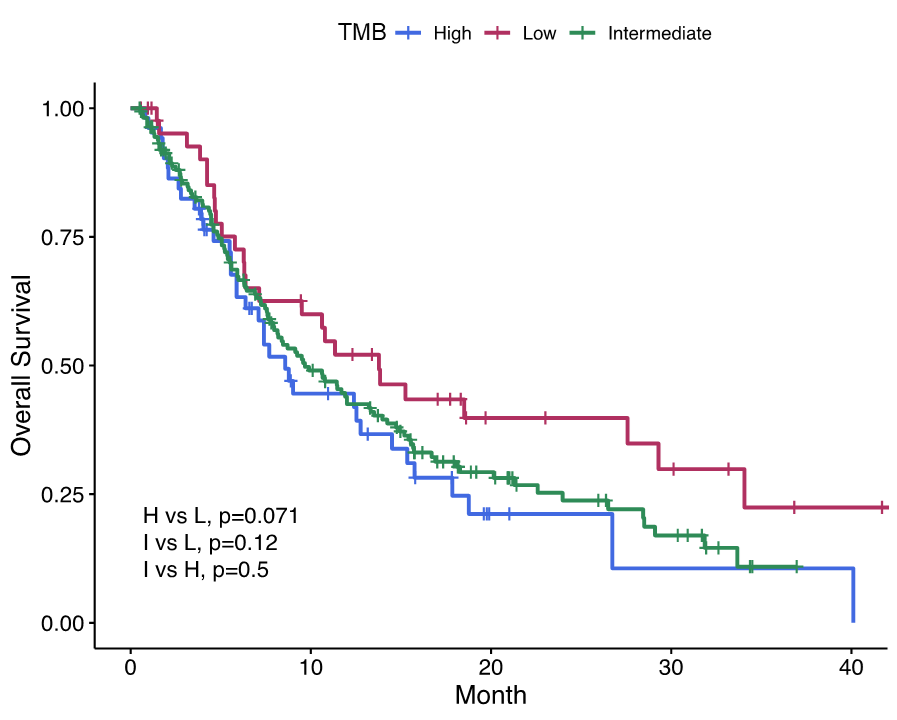
**

**Supplementary Figure 8**

**Supplementary Figure 9**

**Supplementary Figure 10**
